# Same, same, but different: dissimilarities in the hydrothermal germination performance of range-restricted endemics emerge despite microclimatic similarities

**DOI:** 10.1093/conphys/coae009

**Published:** 2024-02-22

**Authors:** Rajapakshe P V G S W Rajapakshe, Sean Tomlinson, Emily P Tudor, Shane R Turner, Carole P Elliott, Wolfgang Lewandrowski

**Affiliations:** ARC Centre for Mine Site Restoration, Curtin University, Bentley, 6102, Australia; School of Molecular and Life Sciences, Curtin University, Bentley, 6102, Australia; Kings Park Science, Department of Biodiversity, Conservation and Attractions, Kings Park, 6005, Australia; School of Molecular and Life Sciences, Curtin University, Bentley, 6102, Australia; School of Biological Sciences, University of Adelaide, Adelaide, 5000, Australia; Kings Park Science, Department of Biodiversity, Conservation and Attractions, Kings Park, 6005, Australia; School of Biological Sciences, University of Western Australia, Crawley, 6009, Australia; ARC Centre for Mine Site Restoration, Curtin University, Bentley, 6102, Australia; School of Molecular and Life Sciences, Curtin University, Bentley, 6102, Australia; Kings Park Science, Department of Biodiversity, Conservation and Attractions, Kings Park, 6005, Australia; School of Biological Sciences, University of Western Australia, Crawley, 6009, Australia; Kings Park Science, Department of Biodiversity, Conservation and Attractions, Kings Park, 6005, Australia; School of Biological Sciences, University of Western Australia, Crawley, 6009, Australia

**Keywords:** Hydrothermal performance, physiological seed dormancy, seed biology, Tetratheca, threatened species conservation

## Abstract

Seed germination responses for most narrow-range endemic species are poorly understood, imperilling their conservation management in the face of warming and drying terrestrial ecosystems. We quantified the realized microclimatic niches and the hydrothermal germination thresholds in four threatened taxa (*Tetratheca erubescens, Tetratheca harperi*, *Tetratheca paynterae* subsp. *paynterae* and *Tetratheca aphylla* subsp. *aphylla*) that are restricted to individual Banded Ironstone Formations in Western Australia. While *T. aphylla* subsp. *aphylla* largely failed to germinate in our trials, all other species demonstrated extended hydrothermal time accumulation (186–500°C MPa days), cool minimum temperatures (7.8–8.5°C), but broad base water potential thresholds (−2.46 to −5.41 MPa) under which germination occurred. These slow germination dynamics are suggestive of cool and wet winter months, where soil moisture is retained to a greater capacity in local microsites where these species occur, rather than the warmer and drier conditions in the surrounding arid environment. Hydrothermal time-to-event modelling showed that each species occupied unique hydrothermal germination niches, which correspond with the microclimatic differences the species are exposed to. Our results provide a baseline understanding for environmental and germination thresholds that govern the recruitment, and ultimately the population structure and persistence, of these short-range endemic plants. In addition, our results can aid future conservation, as well as restoration actions such as translocation to bolster population numbers and to mitigate against losses due to anthropogenic disturbance and global environmental change.

## Teaser

*Tetratheca* spp. are herbaceous shrubs notable for their allopatric speciation over short distances in Western Australia. We compared the germination responses of four species of *Tetratheca* under combined water and temperature stress with the environmental conditions found where these plants grow. Despite similar environments, their germination niches are subtly different.

## Introduction

Rare and threatened species are often considered at most risk of decline, especially when their distribution is range-restricted or geographically isolated, and where significant dispersal barriers exist due to their specific environmental requirements such as unusual substrates, stochastic moisture regimes or extreme conditions between refugia ([Bibr ref7][Bibr ref7], 2019). However, short-range endemic species, those that occur only in very specific habitats ([Bibr ref35][Bibr ref35], 2004), may not be necessarily associated with numerical rarity, nor intrinsic extinction risk ([Bibr ref40][Bibr ref40], 2002). Nevertheless, conserving short-range endemic species is challenging where their only known habitat is vulnerable to anthropogenic alteration ([Bibr ref33][Bibr ref33], 1999), especially where their capacity to disperse in the face of changing conditions is limited. In situations where whole biogeographic regions and taxonomic groups are defined by species with ranges ~10–100 km^2^ ([Bibr ref37][Bibr ref37], 2012), specialization to cryptic environmental conditions is often assumed to drive speciation and persistence of diverse short-range endemic taxa ([Bibr ref35][Bibr ref35], 2004). Under such conditions, investigating the link between biogeography and the environmental conditions that are critical to species' persistence can be particularly revealing of population dynamics, species distributions and management initiatives underpinning conservation and restoration ([Bibr ref44][Bibr ref44], 2018). Understanding species’ physiological thresholds is required to inform management actions, particularly in the face of current global change impacts driven by increasing average temperatures and evaporation, and decreasing rainfall ([Bibr ref51][Bibr ref51], 2021; [Bibr ref49][Bibr ref49], 2022). Moreover, it is important to quantify the impact of environmental change on species' performance at key stages of the life cycle as population persistence can be constrained by ontogenetic-specific requirements ([Bibr ref61][Bibr ref61], 2011; [Bibr ref38][Bibr ref38], 2014; [Bibr ref43][Bibr ref43], 2022).

Seed germination is a crucial stage of the life cycle of plants during which new seedlings are highly susceptible to environmental stressors ([Bibr ref34][Bibr ref34][Bibr ref34], 2016; Gremer, 2023). Recruitment strategies, such as ‘bet-hedging’, where average fitness is sacrificed to minimize variance in fitness, and ‘risk avoidance’, where fitness is maximized by preventing germination in unfavourable conditions, can help mediate against seedling losses by optimizing germination to milder, and more favourable periods for seedling establishment ([Bibr ref2][Bibr ref2][Bibr ref2], 2014; [Bibr ref16][Bibr ref16], 2019; Gremer, 2023). Ecophysiological constraints on germination therefore represent one of the main contributors to plants’ geographical distributions ([Bibr ref19][Bibr ref19], 2017; [Bibr ref55][Bibr ref55][Bibr ref55], 2020), with success or failure at this critical point profoundly impacting all following life stage transitions ([Bibr ref15][Bibr ref15], 2010).

Temperature and water stress are two major environmental factors that regulate the success of seed germination, particularly in water-limited ecosystems ([Bibr ref16][Bibr ref16], 2019). Both temperature and water stress interact to define a bivariate space within which seeds can germinate upon the alleviation of seed dormancy (Bradford, 2002; [Bibr ref41][Bibr ref41], 2018), but quantitative studies accounting for hydrothermal interactions are often understudied for short-range endemics ([Bibr ref46][Bibr ref46], 2020; [Bibr ref51][Bibr ref51], 2021). The hydrothermal time concept establishes thresholds for temperature, as well as moisture availability, that regulate the germination of a seed population over time (Bradford, 2002; [Bibr ref41][Bibr ref41], 2018). Within specific temperature and moisture thresholds, seeds will accumulate hydrothermal time and progress towards germination (Bradford, 2002). The germination limits for temperature are defined between the minimum (*T_b_*, base) and maximum (*T_c_,* ceiling) temperatures, and the base water potential threshold (*Ψ_b_*) for moisture availability (Bradford, 2002). These thresholds are defined by both the dormancy state of the seeds and the specific physiological tolerance to both temperature and water stress for any given species ([Bibr ref1][Bibr ref1][Bibr ref1], 2002; [Bibr ref2][Bibr ref2][Bibr ref2], 2014; [Bibr ref36][Bibr ref36], 2017). Quantifying the hydrothermal germination niche, the hygric and thermal requirements necessary for seed germination, can provide important insights into the roles of these environmental variables in structuring populations and recruitment events, as well as shaping biogeographical patterns of rarity and endemism; critical information when planning future conservation actions such as translocation or assisted migration ([Bibr ref46][Bibr ref46], 2020).

Banded ironstone formations (BIFs) are widely distributed in the semi-arid and arid Yilgarn region of Western Australia, characterized by shallow, skeletal soils, emergent rock surfaces and often substantial topographical variability at small spatial scales ([Bibr ref23][Bibr ref23], 2012). The seeds of range-restricted taxa living on shallow-soil habitats such as outcrop surfaces can have narrower hydrothermal germination norms (i.e. germinate under a narrower range of hygric and/or thermal conditions) compared to widely occurring sympatric species ([Bibr ref15][Bibr ref15], 2010). However, there is a scarcity of data on how closely related short-range endemic species vary in their adaptation to hydrothermal stress, especially where non-overlapping distributions can be quite close together (<50 km) but are separated by different landforms and vegetation communities ([Bibr ref24][Bibr ref24], 2010; [Bibr ref62][Bibr ref62], 2011; [Bibr ref17][Bibr ref17], 2019), essentially forming isolated islands of endemism ([Bibr ref7][Bibr ref7], 2019).

**Figure 1:**
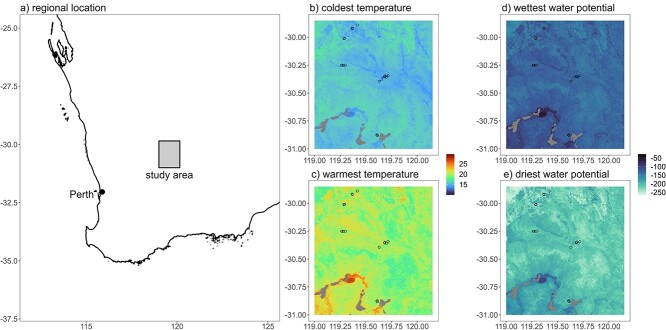
Distributional extents of the *Tetratheca* spp. studied here all fall within an area ~20 × 20 km, located 370 km north-east of Perth, Western Australia (a). The study region is characterized by relatively uniform soil temperatures (b, c) and soil water potentials (d, e). Even in the coolest and wettest periods (b, d) soils in the region are relatively warm and very dry. The points on the maps (b–e) indicate the known populations of our four study species *T. paynterae* subsp. *paynterae*, *T. harperi*, *T. aphylla* subsp. *aphylla* and *T. erubescens* (north to south)*.*

Several studies have highlighted the importance of assessing the role of different environmental variables on the germination response of range-restricted taxa and the implications for their distributional extent ([Bibr ref56][Bibr ref56], 2015; Cochrane, 2020). We quantified the hydrothermal germination niches of a guild of closely related *Tetratheca* Sm. species, each highly restricted (area of occupancy <50km^2^) to geographically separate BIF ranges. Though the species occur on isolated, yet adjacent ranges (Fig. 1), we hypothesized that due to the highly localized range of each species, each species may display subtle differences in their hydrothermal germination envelope that reflects subtle site-specific differences in their microclimate envelope. We also expected the environmental factors most strongly associated with each species’ biogeography to reflect hydrothermal germination niches that are more aligned with cooler and reliably wetter germination windows that reflect the unique capacity for BIFs to retain water for longer periods compared to the surrounding landscape ([Bibr ref7][Bibr ref7], 2019).

## Materials and Methods

### Study species, seed collection and processing

The distributions of *Tetratheca* Sm. (Elaeocarpaceae) species in the semi-arid and arid regions of Western Australia (WA) represents an intriguing display of short-range endemism and diversification, where a number of unique species are confined to rocky habitats for reasons that are not obvious ([Bibr ref14][Bibr ref14][Bibr ref14], 2018; [Bibr ref7][Bibr ref7], 2019). *Tetratheca* species in the region very rarely inhabit more than one outcrop ([Bibr ref62][Bibr ref62], 2011), and neighbouring outcrops often support different species that have been separated for substantial periods of time ([Bibr ref5][Bibr ref5], 2007).

We compared four species of *Tetratheca* that are geographically restricted to BIF habitats in the Yilgarn region of WA in this study (Fig. 1, Supplementary Table S1). The distribution of each species is restricted to one of four BIF ranges (Fig. 1) that are within ~100 km of each other, though separated by different edaphic conditions and floristic communities ([Bibr ref5][Bibr ref5], 2007; [Bibr ref24][Bibr ref24], 2010). *Tetratheca aphylla* F.Muell. subsp. *aphylla* inhabits the relatively deep skeletal soils of lower slopes, hill crests and cliffs of the Helena and Aurora Range ([Bibr ref62][Bibr ref62], 2011). *Tetratheca erubescens* J.P.Bull is found in rock fissures and crevices associated with cliffs, hill crests and steep slopes of the Koolyanobbing Range ([Bibr ref31][Bibr ref31][Bibr ref31], 2019). *Tetratheca harperi* F.Muell. inhabits hill crests, cliffs and cliff slopes of the Mt. Jackson Range ([Bibr ref62][Bibr ref62], 2011). *Tetratheca paynterae* Alford subsp. *paynterae* is restricted to fissures on steep cliffs and tors of the Windarling Range ([Bibr ref32][Bibr ref32], 2019). All four taxa are of high conservation concern and have been gazetted as threatened flora in Western Australia (Herbarium, 1998).

**Figure 2:**
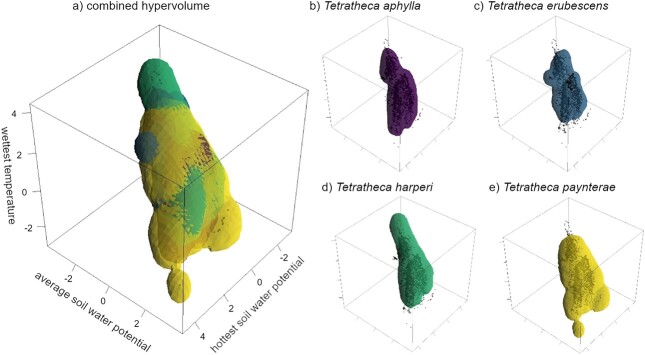
Microclimate hypervolumes constructed using the hydrothermal conditions (average temperature of the wettest quarter; annual average soil water potential; soil water potential of the warmest quarter) of the known occurrence locations for each *Tetratheca* species. The combined hypervolume (a) shows substantial overlap between all four species. Individual hypervolumes (b–e) show more clearly the niche space occupied by each species relative to the constraining parameters. The black points represent the conditions characterizing the known locations of all species combined. The scales on each axis are Euclidean distances of centred and scaled environmental variables.

Seeds were harvested from wild populations of all four species in 2019 (license numbers SW019800 and DRF7789) and once collected were cleaned, processed (Supplementary Table S2) and stored at 15°C and 15% relative humidity (controlled environment room) at the Kings Park Science laboratories, Western Australia for 15 months. These conditions both enhance seed longevity and suppress physiological dormancy loss, maintaining the physiological state of the seeds, including their dormancy status, for several years following collection ([Bibr ref53][Bibr ref53], 2009; [Bibr ref54][Bibr ref54], 2013). Prior to experimentation, seeds were assessed by X-Ray (MX-20 digital X-Ray cabinet, Faxitron, USA), to identify filled seeds. Filled seeds contained fully formed embryos and endosperm, with these anatomical features identified by the presence of uniform white shading of internal tissues. Seeds deviating from this visual appearance were determined to be non-viable and discarded ([Bibr ref18][Bibr ref18][Bibr ref18], 2016).

### Microclimatic conditions of seed sources

The hydrothermal correlates of the realized niche of each study species were quantified using species occurrence data sourced from records maintained by the Herbarium of Western Australia (Herbarium, 1998), supplemented by extensive unpublished surveys by stakeholders from the mineral extraction industry. These known locations were intersected with spatial data describing elevation; aspect and slope ([Bibr ref22][Bibr ref22], 2011; [Bibr ref20][Bibr ref20][Bibr ref20], 2012a; [Bibr ref21][Bibr ref21][Bibr ref21], 2012b); edaphic data describing clay, sand and silt percentage composition [Bibr ref57][Bibr ref57][Bibr ref57][Bibr ref57], 2018b, 2018c) and soil depth interpolated from national soil data provided by the Australian Collaborative Land Evaluation Program (ACLEP), endorsed through the National Committee on Soil and Terrain (NCST; www.clw.csiro.au/aclep). We incorporated these spatial data into soil types using a soil textural triangle ([Bibr ref63][Bibr ref63], 1999) to inform the ‘*micro_global*’ algorithm in the ‘NicheMapR’ package ([Bibr ref30][Bibr ref30][Bibr ref30], 2017) to estimate microclimatic details that are most relevant to seed germination ([Bibr ref50][Bibr ref50], 2020). These specific parameters were chosen to capture the most extreme hydrothermal dynamics experienced by each species, where high water availability at the warmest temperatures may offset thermal stress, and warmer temperatures in the wettest months may promote more rapid germination, given that winter is relatively cold in this region (Supplementary Fig. S1). Higher average soil water availability was thought to represent a less challenging germination environment generally. These data were then averaged between sunrise and sunset at each location to generate daily temperatures and water stress levels 2.5 cm below the soil surface. We used these microclimate estimates to model a 3D environmental niche model built around the annual average soil water potential (SWP), the SWP in the warmest quarter (hottest SWP) and the soil temperature in the wettest quarter (wettest temperature) for each species separately using a Monte Carlo approach, using the ‘*hypervolume*’ function of the ‘*hypervolume*’ R package ([Bibr ref64][Bibr ref64], 2018). The construction function for the hypervolume was a Gaussian kernel density estimation (KDE), where the bandwidth was estimated using a Silverman estimator. The resulting 3D hypervolumes (Fig. 2) were used to define a range of experimental hydrothermal regimes. Hypervolumes of each of the four species were also compared in n-dimensional space to assess overlap using the ‘*hypervolume_overlap_statistics*’ function of the ‘*hypervolume*’ package ([Bibr ref64][Bibr ref64], 2018). The findings of this comparison were used to guide our expectations of likely species hydrothermal performance, in that species with high microclimatic overlap should have highly congruent population performance means ([Bibr ref65][Bibr ref65], 2020).

### Germination requirements and hydrothermal thresholds

Prior to incubation, seeds were surface sterilized with a solution of 2% (w/v) calcium hypochlorite (Ca[OCl]_2_) infused with detergent (Tween 80) for 30 minutes under vacuum (−70 kPa), followed by rinsing with sterile deionized water three times. Seeds were placed inside 90-mm plastic Petri dishes that were lined with moist (9 ml of water per Petri dish) 84-mm germination papers (Advantec, Dublin, CA, USA). The Petri dishes were sealed with plastic wrap to prevent desiccation and covered with aluminium foil to minimize the impact of light, which has a potential confounding effect on germination (Bell, 1994; [Bibr ref48][Bibr ref48], 2018). Before commencing hydrothermal germination experiments, seeds were exposed to a warm stratification treatment that, based on previous research, was assumed to be appropriate for dormancy alleviation in all test species ([Bibr ref17][Bibr ref17], 2019). These seeds were stratified at 30°C for 28 days, then removed from Petri dishes and dried at 15°C and 15% RH (cool dry storage) for 1 week prior to starting the hydrothermal experiments. A random subsample of seeds was also immediately moved from 30°C to 15°C while maintaining their hydration status to confirm the effectiveness of this stratification approach. Seeds were maintained for 4 weeks at 15°C to assess germination after the application of the warm stratification treatment (Supplementary Table S2).

The germination response of seeds to hydrothermal stress was assessed with three replicates of 15 seeds per species, which were incubated at one of 30 hydrothermal stress regimes (water stress levels of 0.0, −0.2, −0.4, −0.6, −0.8 and − 1.2 MPa at temperatures of 10, 15, 20, 22 and 25°C). These hydrothermal regimes encompass a broad range of the hydrothermal conditions constructed for the habitats of the test species (see above; Fig. 2). Seeds were placed on 84-mm germination paper (Advantec, Dublin, CA, USA) moistened with either distilled water or polyethylene glycol 8000 (PEG) solution (of different concentrations) to generate the range of water potentials outlined above for each incubation temperature (Michel, 1983). These solutions had 2% (v/v) PPM (Plant Preservation Mixture, Austratec Pty Ltd, Bayswater, Victoria Australia) added to minimize microbial contamination during stratification and incubation treatments. Nine millilitres of this solution was added per 90-mm Petri dish for all species, except Petri dishes containing *T. aphylla* subsp. *aphylla*. These were instead irrigated with the addition of 1 μM karrikinolide (KAR_1_—[Bibr ref67][Bibr ref67][Bibr ref67]; [Bibr ref68][Bibr ref68][Bibr ref68]) for the duration of the incubation period as preliminary data found an improved germination response for this species following the application of KAR_1_ (Supplementary Table S2). All Petri dishes were sealed with plastic wrap and aluminium foil. During the incubation period, iButton data loggers (Maxim Integrated™, San Jose, USA) were placed in the middle of each set of Petri dishes, which were subjected to the same treatment regime to record the temperature that seeds were exposed to within each incubator while the germination trial was underway (Supplementary Table S3). Germination was identified when the radicle emerged >2 mm from the testa and was scored 3 days per week for 56 days.

### Statistical analysis

#### Germination modelling

The germination responses of each species, except *T. aphylla* subsp. *aphylla*, which failed to germinate beyond 27% in any given treatment, were characterized by a hydrothermal time-to-event (HTTE) model ([Bibr ref41][Bibr ref41][Bibr ref41]). HTTE models are well-grounded in physiological theory and work on the assumption that germination does not occur outside certain temperature or water stress thresholds and parameterize the hydrothermal germination niche by a function of time ([Bibr ref41][Bibr ref41][Bibr ref41]). Preliminary assessments of a global model compared five defined HTTE models with selection based on graphical inspection of goodness of fit and Akaike’s Information Criterion (AIC; Akaike(1974)). The model selected was a six-parameter log-logistic cumulative distribution function (HTTEM; [Bibr ref39][Bibr ref39], 2017, [Bibr ref41][Bibr ref41], 2018, [Bibr ref42][Bibr ref42], 2022), defined as:

\begin{align*} P\left(t,\varPsi\!, T\right)\!=\!\varPhi \left[b\left(\varPsi\!, T\right)\!,{Pmax}_{HTTE}\left(\varPsi\!, T\right)\!, GR{50}_{HTTE}\left(\varPsi\!, T\right)\right]\\ Eqn.1 \end{align*}

With three distinct sub-models:

$$ GR{50}_{HTTE}\left(T,\varPsi \right)=\frac{T-{T}_b}{\theta HT}\left[\varPsi -{\varPsi}_b-k\left(T-{T}_b\right)\right]\ Eqn.1.1 $$

$$ {Pmax}_{HTTE}\left(T,\varPsi \right)=G\left\{1-{e}^{\left[\ \frac{\big(\varPsi -{\varPsi}_b-k\big(T-{T}_b\big)\big)}{\sigma_{\varPsi_b}}\right]}\right\}\ Eqn.1.2 $$

$$ {F}_{HTTE}\left(t,T,\varPsi \right)\!=\!\frac{Pmax_{HTTE}}{1\!+\!{e}^{\left(b\left[\log (t)-\log \left({}^{1}\!\left/ \!{}_{GR{50}_{HTTE}}\right.\right)\right]\right)}}\ Eqn.1.3 $$

**Table 1:** Patterns of niche overlap between pairs of *Tetratheca* species. The Sorenson overlap indicates the proportion of the combined niche space that the two species share, while the Fraction Unique columns indicate what proportion of that combined niche space is occupied by only the indicated species (Sp. 1 or Sp. 2)

**Species 1**	**Species 2**	**Sorensen overlap**	**Fraction unique (Sp. 1)**	**Fraction unique (Sp. 2)**
*T. aphylla*	*T. erubescens*	0.64	0.45	0.23
	*T. harperi*	0.66	0.18	0.44
	*T. paynterae*	0.70	0.10	0.43
				
	*T. harperi*	0.56	0.15	0.58
*T. erubescens*	*T. paynterae*	0.55	0.11	0.60
				
*T. harperi*	*T. paynterae*	0.70	0.27	0.33

Where germination proportion is dependent upon time (*t*), temperature (*T*) and base water potential *(Ψ)*; the model is computed by the reciprocal of time to the 50th percentile of the germinated fraction ($\frac{1}{t_g}$) *GR*50*_HTTE_,* maximum germination proportion (*G*; while accounting for possibility that *Pmax_HTTE_* may not reach 1 under any condition); median base water potential limiting to germination in the seed lot (*Ψ_b_*); variability of median base water potential (${\sigma}_{\varPsi_b}$); base or minimum temperature for germination (*T_b_*); hydrothermal time constant (*θHTT*; MPa °C days; the hydrothermal time required for germination in the seed population), slope of the linear increase in *Ψ_b_* when temperature increases (*K_t_*); and the slope of the germination curve in the *F_HTTE_* distribution (*b*). The model is only defined under the conditions between which germination occurs (i.e. between *T_b_* and *T_c_*; maximum temperature for germination) and is equal to 0 for temperatures *T*$\le$*T_b_* or *T*$\ge$*T_c_*, and water potentials $\varPsi$ < ${\varPsi}_b-k\left(T-{T}_b\right)$. Models were fitted using the *drcte* function of the ‘*drcte*’ statistical package ([Bibr ref42][Bibr ref42], 2022) with hydrothermal time to event model coded in the *‘drcSeedGerm*’ package ([Bibr ref42][Bibr ref42], 2022) using the R statistical environment (version 4.2.3) and RStudio Version 2023.03.0 ([Bibr ref45][Bibr ref45][Bibr ref45][Bibr ref45], 2022).

After global model selection, we constructed a species-specific model, and determined if the delineation identified responses that were significantly different to the ‘global model’ via an F-test using the *anova* function in the ‘*stats*’ package ([Bibr ref45][Bibr ref45][Bibr ref45][Bibr ref45], 2022). *Post hoc* comparisons of parameter estimates were conducted with the *compParm* function in the ‘*drc*’ package ([Bibr ref47][Bibr ref47][Bibr ref47], 2012) with significance established at *P* < 0.05. However, we do not report on comparisons for *b,* as it is a shape parameter that is regarded as independent from the environmental covariates, or ${\sigma}_{\varPsi_b}$*,* due to insignificant fit and parameterization in the global model. After the hydrothermal time distributions were modelled, using equation 1.3, hydrothermal germination responses were plotted at 15, 30 and 60 days to quantify the changes in germination behaviour for each species. Maximum germination proportions (*G_max_*) and thresholds for *T_b_* and *T_c_*, as well as *Ψ_b_* were derived from the predictions at each increment, defining the breadth of the hydrothermal germination niche as it changes over time.

## Results

### Niche overlap in microclimatic conditions

The highest degree of microclimatic niche overlap (70%) was between *T. paynterae* subsp. *paynterae* and *T. aphylla* subsp. *aphylla* and between *T. paynterae* subsp. *paynterae* and *T. harperi*, with all four species sharing at least 50% of their 3D microclimatic niche space with each other (Table 1; Fig. 2). The percentage of unique space a species had was complex, as different species nested within each other’s realized niches in different ways. The main patterns were that *T*. *erubescens* consistently had the lowest percentage of unique space (11–23%) compared to the other three (i.e. had a realized niche that mostly nested within the other three). This was followed by *T. aphylla* subsp. *aphylla* (10–18%) compared to *T. paynterae* subsp. *paynterae* and *T. harperi*, except when compared to *T*. *erubescens*. Compared to *T*. *erubescens*, *T. aphylla* subsp. *aphylla* had a higher percentage (45%) of unique space that mainly related to having a broader envelope of soil temperature in the wettest quarter (wettest temperature), where the known occurrence locations are found. Leaving *T. paynterae* subsp. *paynterae* and *T. harperi* to both retain ~30% of the combined niche space uniquely to themselves (Table 1), that related to also having a broader microclimatic envelope across all three hydrothermal conditions, albeit both dominating the opposite ends of this envelope. *Tetratheca harperi* uniquely occupied microclimates characterized by higher temperatures than any other species, while *T. paynterae* subsp. *paynterae* occupied microclimates characterized by the coolest and wettest conditions (Fig. 2).

### Hydrothermal time-to-event germination modelling

The global HTTE model resolved significant fits for each parameter (*P* < 0.001), excluding ${\sigma}_{\varPsi_b}$*.* As such, we conducted no further interrogation or *post hoc* comparison of ${\sigma}_{\varPsi_b}$. The global model had an AIC index of 16 052.54 and log-likelihood of −2153.27. The species-specific model significantly improved model parsimony and reduced the AIC to 15 769.0 and log-likelihood of −2025.75 (F_14,5859_ = 26.34, *P* < 0.001), indicating species-level differences in hydrothermal performance.

Species-level differences in hydrothermal performance occurred for all parameters excluding ${\sigma}_{\varPsi_b}$ and *b* (Table 2). The projected mean maximum germination (*G_max_*) proportion was significantly lower for *T. paynterae* subsp. *paynterae* (0.21 ± 0.03) than *T. erubescens* (0.64 ± 0.16; t = 2.18; *P* = 0.02) or *T. harperi* (0.56 ± 0.03; t = 3.15; *P* = 0.002). However, *T. paynterae* subsp. *paynterae* required significantly less hydrothermal time (186.8 ± 34.1 MPa °C days) to reach maximum germination compared to *T. erubescens* (421.4 ± 40.5 MPa °C days; t = 2.25; *P* = 0.006) or *T. harperi* (499.9 ± 46.3 MPa °C days; t = 3.05; *P* = 0.002). Base temperature for germination (*T_b_*) was significantly lower in *T. harperi* (7.82 ± 0.22°C) compared to *T. erubescens* (8.50 ± 0.17°C; t = 2.21; *P* = 0.027), but statistically indistinguishable from *T. paynterae* subsp. *paynterae* (8.51 ± 0.45°C; t = −1.45; *P* = 0.145). There were significant differences between *K_t_* parameter estimates only between *T. erubescens* (0.34 ± 0.04) and *T. paynterae* subsp. *paynterae* (0.16 ± 0.02; t = 3.01; *P* = 0.002). Lastly, median base water potential was significantly less negative (i.e. requires more water availability) in *T. paynterae* subsp. *paynterae* (−2.47 ± 0.18 MPa) compared to both *T. erubescens* (−5.41 ± 0.57 MPa; t = 4.19; *P* < 0.001) and *T. harperi* (−4.61 ± 0.54 MPa; t = 3.34; *P* < 0.001).

**Table 2:** Hydrothermal time parameters of *T. erubescens*, *T. harperi* and *T. paynterae* subsp. *paynterae*. Population differences for HTTE-parameters are summarized with the F-value, and specific parameter comparisons summarized with letters in superscript (at *P* < 0.05). *Tetratheca aphylla* subsp. *aphylla* did not germinate in this study

***Parameter***	***T. erubescens***	***T. harperi***	***T. paynterae***
*G*	0.65 ± 0.16 ^a^	0.56 ± 0.03 ^a^	0.21 ± 0.04 ^b^
_b_ [MPa]	−5.41 ± 0.57^a^	−4.61 ± 0.54 ^a^	−2.46 ± 0.19 ^b^
Kt	0.34 ± 0.05 ^a^	−0.24 ± 0.04 ^ab^	−0.16 ± 0.02 ^b^
Tb [°C]	8.51 ± 0.45 ^a^	7.82 ± 0.23 ^b^	8.51 ± 0.45 ^ab^
σ	1.21 ± 0.76 ^a^	0.14 ± 0.34 ^a^	0.09 ± 0.12 ^b^
*θ*HTT [MPa °C days]	421 ± 40.5 ^a^	500 ± 46.3 ^b^	186 ± 34.1 ^b^
b	4.99 ± 0.38 ^a^	6.81 ± 0.57 ^a^	3.78 ± 0.61 ^b^

Population difference F-value: 26.34^***^

### Hydrothermal threshold variation over a 60-day window

**Figure 3:**
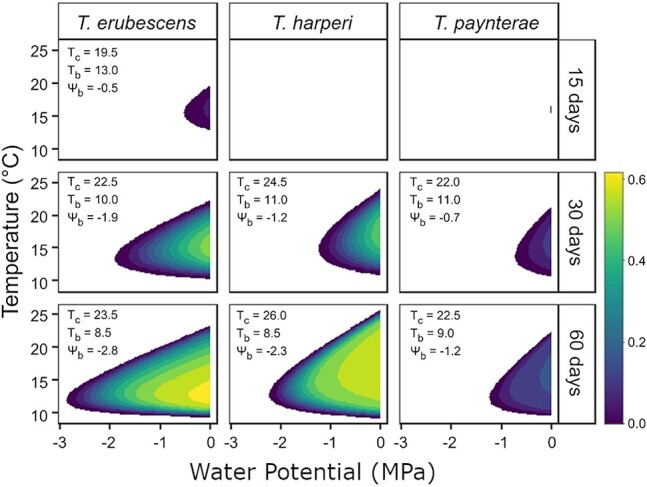
Modelled hydrothermal germination windows of *T. erubescens*, *T. harperi* and *T. paynterae* subsp. *paynterae* for 15, 30 and 60 days across a simulated temperature, water potential gradient. Colour scale bar represents germination proportion.

Maximum germination (*G_max_*) proportion after a 15-day window was 0.12 in *T. erubescens*, in contrast to 0.04 and 0.05 in *T. harperi,* and *T. paynterae* subsp. *paynterae*, respectively. These proportions increased progressively in all species, although variation was characterized by overall lower germination performance in *T. paynterae* subsp. *paynterae* (*G_max_* < 0.21, after 60 days), in contrast to *T. erubescens* and *T. harperi* (*G_max_* > 0.5 after 60 days). The lower germination performance of *T. paynterae* subsp. *paynterae* was also matched by the narrowest hydrothermal ranges for germination in *T. paynterae* subsp. *paynterae* (see *T_b_, T_c_* and *Ψ_b_*, Fig. 3). While all species were constrained to germinate between 10 and 25°C, *T. harperi* had the broadest range over time (Fig. 3). There was progressive broadening of *Ψ_b_* thresholds in all species, with *T. erubescens* characterized by the lowest *Ψ_b_* over the time-course (refer to Fig. 3 for all *T_b_, T_c_* and *Ψ_b_* parameter estimates).

## Discussion

In natural environments, successful seedling establishment is reliant on the duration of the window of opportunity for germination (Gremer, 2023). Hydrothermal stress is a major selective pressure that restricts that window of opportunity, particularly in semi-arid ecosystems where water availability can be extremely variable from year to year due to the dynamic nature of these extreme environments ([Bibr ref28][Bibr ref28], 2016). To our knowledge, our results are the first to characterize the hydrothermal germination niche for *Tetratheca* species, using a contemporary HTTE modelling approach ([Bibr ref41][Bibr ref41], 2018). The cool thermal thresholds quantified from our hydrothermal time modelling are consistent with reports in the region ([Bibr ref13][Bibr ref13], 2022) that all four species tend to germinate during the cooler winter months, and the HTTE models explained slowed germination, along with a gradual widening of hydrothermal thresholds over a 60-day germination window. Despite substantial overlap in the realized microclimatic niche estimated from known occurrence locations, subtle interspecific differences were evident in the hydrothermal niche consistent with the specific biogeography of each species.

### Microclimatic niche and germination strategy

The high degree of overlap between the microclimatic niches and the hydrothermal optima of the *Tetratheca* species (Fig. 2, Fig. 3) suggests that similar selection pressures have optimized their hydrothermal niches, despite their geographic and genetic isolation ([Bibr ref7][Bibr ref7], 2019). The locations where these species occur are less stressful at critical times by comparison to the surrounding landscape, with SWPs higher than −1.2 MPa and soil temperatures between 5 and 25°C occur during a potential germination window over the winter rainfall period for the region (Supplementary Figure S1). As demonstrated by the HTTE models, the three modelled *Tetratheca* species require long periods of hydrothermal time accumulation to germinate, with germination constrained to comparatively cooler and wetter conditions (Table 2; Fig. 3).

Despite the narrow temperature range (10–25°C), all species included in the analysis demonstrated remarkably wide base water potentials for germination, suggesting that a proportion of the seed population may be capable of germinating into significant water stress. Collectively, these observations suggest that different outcrop-endemic *Tetratheca* species may be differently adapted to cope with limited water availability. This interpretation is at odds with that which may be drawn when considering the high degrees of microclimatic niche overlap between the species (see above). However, despite the similarities present in the microclimatic niche, each species inhabits some unique microclimatic niche space, which correspond with their dissimilarities in hydrothermal niches. For example, *T. erubescens* inhabits the core of the microclimate envelope shared by all species, defined by moderately broad thermal conditions and SWPs (Fig. 2), and germinates relatively well across a wide combination of water stress and thermal stress (Fig. 3). It is also the youngest species in a phylogenetic sense ([Bibr ref7][Bibr ref7], 2019), and thus may not have specialized to microclimatic conditions unique to Koolyanobbing Range. Where *T. harperi* occurs uniquely, this microclimatic space is principally defined by warmer temperatures (Fig. 2), and the species has the broadest tolerance to thermal stress of any of the species studied (Fig. 3). Finally, *T. paynterae* subsp. *paynterae* occurs more in cooler and wetter microclimate space than any of the other species (Fig. 2) and has the lowest germination success of the three species that we characterized here, and only at cooler temperatures and lower water stress compared to either *T. harperi* or *T. erubescens*. The more limited germination success could be attributed to seeds of *T. paynterae* subsp. *paynterae* that remained in a conditional dormancy state at the time of the germination study, which explains the limited and narrow optimal conditions for germination, in contrast to the other species ([Bibr ref2][Bibr ref2][Bibr ref2], 2014).

By capturing the time element of the germination window, we have shown differences in how divergent species respond to ephemeral conditions. The modelled *Tetratheca* species require hydrothermal time accumulation between 186 and 500 MPa °C days to complete germination of the seed population, which could be attributed to dormant proportions in the seed cohort ([Bibr ref2][Bibr ref2][Bibr ref2], 2014), or being intrinsically slow-germinating species. Slow germination has been reported in many taxa endemic to mesic habitats of Western Australia ([Bibr ref56][Bibr ref56], 2015; Cochrane, 2020; [Bibr ref13][Bibr ref13], 2022). However, slow germination is potentially risky as it could compromise seedling survival in microhabitats if they fail to retain moisture for a sufficient period following rainfall episodes. This supports contentions that the study species may have contracted to microhabitats on outcrops that can retain high soil moisture for sufficient time to support germination beyond what would normally be expected in surrounding environments ([Bibr ref24][Bibr ref24], 2010; [Bibr ref62][Bibr ref62], 2011). The extended hydrothermal germination requirement may be a common characteristic of floristic endemism in the inselberg communities of the Yilgarn region ([Bibr ref23][Bibr ref23], 2012), and likely influences the biogeography of other BIF-restricted species such as *Ricinocarpos brevis*, *Darwinia masonii* and *Lepidosperma gibsonii* ([Bibr ref9][Bibr ref9]). While the hydrothermal requirements for seed germination are largely unexplored for many BIF-restricted species, understanding these dynamics could provide critical insight into the constraints and requirements driving recruitment events in these floristically diverse ecosystems.

### Seed dormancy and ‘bet-hedging’

The specialization of *Tetratheca* species to slow germination in relatively cool and wet conditions is also likely enhanced by the presence of physiological dormancy, as seeds are cued for germination via warm stratification as soil temperatures gradually drop, leading into the cooler relatively wetter winter season ([Bibr ref70][Bibr ref70], 2006; [Bibr ref29][Bibr ref29], 2023). Previous studies on several BIF-endemic species suggest that in physiologically dormant, range-restricted species, the windows of opportunities for seedling establishment are rare, unpredictable and perhaps only occur every few years ([Bibr ref62][Bibr ref62], 2011; [Bibr ref17][Bibr ref17], 2019). Although not an essential trait in the formation of a soil seedbank ([Bibr ref25][Bibr ref25], 2020), dormant seeds are likely to persist in the soil seedbank while conditions are not conducive to germination and seeding recruitment, and seed dormancy can leave ungerminated or dormant seeds exposed to long periods between recruitment events ([Bibr ref71][Bibr ref71], 2015; [Bibr ref72][Bibr ref72], 2019). Indeed, warm stratification is a relatively uncommon dormancy release treatment ([Bibr ref29][Bibr ref29], 2023), allowing seeds to retain a slow germination pattern in cool moist conditions as gradual dormancy release occurs in response to an initial warmer, moist soil environment. This gradual drop in soil temperature acts as an initial environmental filter to cue seeds for seasonal germination, rather than opportunistic, aseasonal germination ([Bibr ref56][Bibr ref56], 2015). As such, any given hydrothermal window could stimulate the germination of only a proportion of the available soil seed bank, minimizing the risk of unsuccessful germination in dynamic and unpredictable environments. While we cannot conclusively demonstrate this for the *Tetratheca* species studied here, other short-range endemic species in the region, such as *Darwinia masonii* and *Lepidosperma gibsonii* possess physiological seed dormancy and form long-lived soil seed banks ([Bibr ref17][Bibr ref17], 2019; [Bibr ref72][Bibr ref72], 2019). The mechanisms underpinning the alleviation of seed dormancy under natural conditions are often difficult to unravel ([Bibr ref73][Bibr ref73], 2007), and additional research is necessary to understand the nuances of dormancy loss and germination stimulation in these species more broadly.

### Limitations to model interpretations

While the applied dormancy alleviation treatments resulted in varied degrees of germination success across the studied species, the low germination rates seen for *T. aphylla* subsp. *aphylla* may be a result of physiological dormancy that was not adequately overcome. This suggests that an extended stratification period may be required, in addition to the stimulus applied (KAR_1_). Moreover, the germination responses here may still encapsulate some component of seed dormancy, which would account for the generally much lower germination success that we also found for *T. paynterae* subsp. *paynterae* across all hydrothermal conditions (Fig. 3). In general, exposure to KAR_1_ enhanced the germination success of these species, potentially indicating that exposure to smoke represents a critical environmental stimulus that defines the germination requirements of those taxa, particularly under conditions where disturbance events (e.g. fire) may occur ([Bibr ref52][Bibr ref52], 2018). As such, dormancy alleviation and the hydrothermal niche require further attention to provide a comprehensive understanding of the drivers of *in situ* recruitment beyond the fundamental microclimatic elements that we have reported here.

The capacity of range-restricted taxa to persist in the face of changing conditions is dependent on the influence of interacting niche dimensions on population demographics ([Bibr ref44][Bibr ref44], 2018). Our study captured the performance norms of seeds collected under current climatic conditions but offers little direct insight into phenotypic flexibility in these norms. The extent to which climate change can affect the physiological limits and optima of range-restricted taxa in terms of altered rainfall patterns and temperature, as well as dormancy cycling, must also be quantified ([Bibr ref60][Bibr ref60], 2011; [Bibr ref12][Bibr ref12], 2014; Cochrane, 2017). However, a greater suite of species should be compared including both range-restricted taxa as well as those with wide distributions to gain useful insights into how hydrothermal germination niche affects patterns of species distribution. Collectively, this can help determine whether range-restricted species display attributes not typically observed with more broadly occurring sympatric species (Byrne, 2019).

### Conservation implications

This study highlights the significant role of hydrothermal stress on germination responses that are likely to influence population dynamics and distribution patterns of BIF specialist *Tetratheca* species. The high degree of overlap between the microclimatic niches, coupled with existing phylogenetic evidence, supports suggestions that these are allopatric species (i.e. localized speciation on extended time frames; [Bibr ref7][Bibr ref7][Bibr ref7]) that have persisted on localized outcrops due to the relatively milder conditions they support compared to the surrounding semi-arid landscapes ([Bibr ref7][Bibr ref7], 2018). The short-range endemic *Tetratheca* species are associated with such habitats ([Bibr ref5][Bibr ref5], 2007; [Bibr ref7][Bibr ref7], 2018), potentially because of a conserved germination strategy that is more aligned with cooler and reliably wetter regions in southwest Western Australia. This conserved germination strategy suggests that *Tetratheca* species endemic to BIFs may be more susceptible to environmental changes as the climate further warms and dries. Furthermore, other *Tetratheca* species endemic to granitic, sandstone and ironstone inselbergs throughout WA have unknown germination requirements, leaving uncertainties as to how they may respond to the unpredictable changes of climate likely to eventuate throughout the arid ecosystems of Australia (Lioubimtseva, 2004).

BIFs in the region are subject to extensive mineral exploitation ([Bibr ref24][Bibr ref24], 2010). Management actions in response to mining disturbance often involve mitigation translocations (*sensu*[Bibr ref75][Bibr ref75][Bibr ref75]) to establish insurance populations. Underpinning these approaches is the recognition that there is often ‘uninhabited’ niche space in the distribution of short-range endemic flora ([Bibr ref7][Bibr ref7], 2019), and that rapidly identifying this provides suitable recipient locations for translocated individuals. The data that we report here suggest that, while the *Tetratheca* species show high overlap in the microclimatic niches in which they occur, subtle interspecific differences in their germination performance norms are consistent with regions of the microclimatic niche that are uniquely occupied by each species. From the perspective of planning and executing mitigation translocations, while vacant habitats may appear suitable to *Tetratheca* spp. in a broad sense, the mechanistic drivers underpinning this vacancy can be complex, subtle and species-specific. Thus, introducing individuals into habitats without capturing these subtleties may result in suboptimal population performance in terms of form, function and fitness, thereby reducing long-term translocation success. Consequently, we believe that greater success in mitigation translocations is likely to result from greater investment in unravelling these critical drivers.

## Supplementary Material

Web_Material_coae009

## Data Availability

The raw data underpinning the germination trials are provided as supplemental material. The location data underpinning the biogeographical analyses cannot be released for legislative reasons protecting rare species in Western Australia.

## References

[ref69] Akaike H (1974) A new look at the statistical model identification. IEEE Transactions on Automatic Control 19: 716–723.

[ref1] Alvarado V , Bradford KJ (2002) A hydrothermal time model explains the cardinal temperatures for seed germination. Plant, Cell and Environment 25: 1061–1069. 10.1046/j.1365-3040.2002.00894.x.

[ref2] Baskin CC , Baskin JM (2014) Seeds: Ecology, Biogeography, and Evolution of Dormancy and Permination, Ed2nd. Elsevier Academic Press, New York

[ref3] Bell DT (1994) Interaction of fire, temperature and light in the germination response of 16 species from the eucalyptus marginata forest of South-Western Australia. Australian Journal of Botany 42: 501–509. 10.1071/BT9940501.

[ref64] Blonder B , Morrow CB, Maitner B, Harris DJ, Lamanna C, Violle C, Enquist BJ, Kerkhoff AJ (2018) New approaches for delineating n-dimensional hypervolumes. Methods in Ecology and Evolution 9: 305–319.

[ref4] Bradford KJ (2002) Applications of hydrothermal time to quantifying and modeling seed germination and dormancy. Weed Science 50: 248–260. 10.1614/0043-1745(2002)050[0248:AOHTTQ]2.0.CO;2.

[ref75] Bradley H , Tomlinson S, Craig MD, Cross AT, Bateman PW (2020) Mitigation translocation as a management tool. Conservation Biology 36: e13667. 10.1111/cobi.13667 33210780

[ref5] Butcher R , Byrne M, Crayn DM (2007) Evidence for convergent evolution among phylogenetically distant rare species of *tetratheca* (elaeocarpaceae, formerly tremandraceae) from Western Australia. Australian Systematic Botany 20: 126–138. 10.1071/SB06017.

[ref6] Byrne M (2019) Genetics and ecology of plant species occurring on the banded iron formations in the yilgarn, Western Australia. Australian Journal of Botany 67: 165–171. 10.1071/BT19048.

[ref7] Byrne M , Krauss SL, Millar MA, Elliott CP, Coates DJ, Yates C, Binks RM, Nevill P, Nistelberger H, Wardell-Johnson G et al. (2019) Persistence and stochasticity are key determinants of genetic diversity in plants associated with banded iron formation inselbergs. Biol Rev 94: 753–772. 10.1111/brv.12477. 30479069

[ref9] Byrne M , Yeates D, Joseph L, Kearney M, Bowler J, Williams M, Cooper S, Donnellan S, Keogh J, Leys R (2008) Birth of a biome: insights into the assembly and maintenance of the Australian arid zone biota. Mol Ecol 17: 4398–4417. 10.1111/j.1365-294X.2008.03899.x. 18761619

[ref65] Carscadden KA , Emery NC, Arnillas CA, Cadotte MW, Afkhami MW, Afkhami ME, Gravel D, Livingstone SW, Wiens JJ (2020) Niche breadth: causes and consequences for ecology, evolution, and conservation. The Quarterly Review of Biology 95: 179–214.

[ref68] Chiwocha SD , Dixon KW, Flematti GR, Ghisalberti EL, Merritt DJ, Nelson DC, Riseborough JAM, Smith SM, Stevens JC (2009) Karrikins: a new family of plant growth regulators in smoke. Plant Science 177: 252–256.

[ref10] Cochrane A (2017) Modelling seed germination response to temperature in eucalyptus l'her. (myrtaceae) species in the context of global warming. Seed Sci Res 27: 99–109. 10.1017/S0960258517000010.

[ref11] Cochrane A (2020) Temperature thresholds for germination in 20 short-range endemic plant species from a greenstone belt in southern Western Australia. Plant Biology 22: 103–112. 10.1111/plb.12951. 30556244

[ref12] Cochrane A , Hoyle GL, Yates CJ, Wood J, Nicotra AB (2014) Predicting the impact of increasing temperatures on seed germination among populations of Western Australian banksia (proteaceae). Seed Sci Res 24: 195–205. 10.1017/S096025851400018X.

[ref13] Dalziell EL , Lewandrowski W, Commander LE, Elliott CP, Erickson TE, Tudor EP, Turner SR, Merritt DJ (2022) Seed traits inform the germination niche for biodiverse ecological restoration. Seed Science and Technology 50: 103–124. 10.15258/sst.2022.50.1.s.06.

[ref14] Di Virgilio G , Wardell-Johnson G, Robinson T, Temple-Smith D, Hesford J (2018) Characterising fine-scale variation in plant species richness and endemism across topographically complex. semi-arid landscapes 156: 59–68. 10.1016/j.jaridenv.2018.04.005.

[ref15] Donohue K , de Casas RR, Burghardt L, Kovach K, Willis CG (2010) Germination, postgermination adaptation, and species ecological ranges. Annu Rev Ecol Evol Syst 41: 293–319. 10.1146/annurev-ecolsys-102209-144715.

[ref16] Duncan C , Schultz N, Good M, Lewandrowski W, Cook S (2019) The risk-takers and avoiders: germination sensitivity to water stress in an arid zone with unpredictable rainfall. AoB Plants 11: plz066. 10.1093/aobpla/plz066. 31777652 PMC6863470

[ref17] Elliott CP , Lewandrowski W, Miller BP, Barrett M, Turner SR (2019) Identifying germination opportunities for threatened plant species in episodic ecosystems by linking germination profiles with historic rainfall events. Australian Journal of Botany 67: 256–267. 10.1071/BT18215.

[ref18] Erickson TE , Merritt DJ (2016) Seed collection, cleaning, and storage procedures. In Erickson TE, Barrett R, Merritt DJE, Dixon KW, eds, ‘Pilbara Seed Atlas and Field Guide: Plant Restoration in Australia's Arid Northwest'. CSIRO Publishing, Melbourne

[ref19] Fernández-Pascual E , Pérez-Arcoiza A, Prieto JA, Díaz TE (2017) Environmental filtering drives the shape and breadth of the seed germination niche in coastal plant communities. Ann Bot 119: 1169–1177. 10.1093/aob/mcx005. 28334139 PMC5604583

[ref67] Flematti GR , Ghisalberti EL, Dixon KW, Trengove RD, Flematti GR (2004) A compound from smoke that promotes seed germination. Science 305: 977–977. 15247439 10.1126/science.1099944

[ref20] Gallant JC , Austin J (2012a) Aspect derived from 1 srtm dem-s v6. CSIRO Data Collection . 10.4225/08/56D778315A62B.

[ref21] Gallant JC , Austin J (2012b) Slope derived from 1 srtm dem-s. V4. CSIRO Data Collection . 10.4225/08/5689DA774564A.

[ref22] Gallant JC , Dowling TI, Read AM, Wilson N, Tickle P, Inskeep C (2011) 1 second srtm derived digital elevation models user guide. Geoscience Australia, Canberra, Australia

[ref63] Gerakis A , Baer B (1999) A computer program for soil textural classification. Soil Science Society of America Journal 63: 807–808

[ref23] Gibson N , Meissner R, Markey AS, Thompson WA (2012) Patterns of plant diversity in ironstone ranges in arid south western Australia. J Arid Environ 77: 25–31. 10.1016/j.jaridenv.2011.08.021.

[ref24] Gibson N , Yates CJ, Dillon R (2010) Plant communities of the ironstone ranges of south western Australia: hotspots for plant diversity and mineral deposits. Biodivers Conserv 19: 3951–3962. 10.1007/s10531-010-9939-1.

[ref25] Gioria M , Pyšek P, Baskin CC, Carta A (2020) Phylogenetic relatedness mediates persistence and density of soil seed banks. J Ecol 108: 2121–2131. 10.1111/1365-2745.13437.

[ref26] Gremer JR (2023) Looking to the past to understand the future: linking evolutionary modes of response with functional and life history traits in variable environments. New Phytol 237: 751–757. 10.1111/nph.18605. 36349401

[ref27] Herbarium WA (1998) Florabase – The Western Australian Flora. Department of Parks and Wildlife, Perth, W.A

[ref28] Huang Z , Liu S, Bradford KJ, Huxman TE, Venable DL (2016) The contribution of germination functional traits to population dynamics of a desert plant community. Ecology 97: 250–261. 10.1890/15-0744.1. 27008793

[ref29] Just M , Cross AT, Lewandrowski W, Turner SR, Merritt DJ, Dixon K (2023) Seed dormancy alleviation by warm stratification progressively widens the germination window in Mediterranean climate rutaceae. Australian Journal of Botany 71: 55–66. 10.1071/BT22076.

[ref30] Kearney MR , Porter WP (2017) Nichemapr–an r package for biophysical modelling: the microclimate model. Ecography 40: 664–674. 10.1111/ecog.02360.

[ref31] Krauss SL , Anthony JM (2019) The potential impact of mining on population genetic variation in the banded ironstone formation endemic tetratheca erubescens (elaeocarpaceae). Australian Journal of Botany 67: 172. 10.1071/BT18054.

[ref32] Ladd PG , Yates CJ, Dillon R, Palmer R (2019) Pollination ecology of tetratheca species from isolated, arid habitats (banded iron formations) in Western Australia. Australian Journal of Botany 67: 248. 10.1071/BT18249.

[ref33] Lande R , Landweberg LF, Dobson AP (1999) Extinction risks from anthropogenic, ecological, and genetic factors. In LF Landweberg, AP Dobson, eds, Genetics and the Extinction of Species: DNA and the Conservation of Biodiveristy. Princeton Univesity Press, Princeton, New Jersey, pp. 1–22

[ref34] Larson JE , Funk JL (2016) Regeneration: an overlooked aspect of trait-based plant community assembly models. J Ecol 104: 1284–1298. 10.1111/1365-2745.12613.

[ref35] Lavergne S , Thompson JD, Garnier E, Debussche M (2004) The biology and ecology of narrow endemic and widespread plants: a comparative study of trait variation in 20 congeneric pairs. Oikos 107: 505–518. 10.1111/j.0030-1299.2004.13423.x.

[ref36] Lewandrowski W , Erickson TE, Dixon KW, Stevens JC (2017) Increasing the germination envelope under water stress improves seedling emergence in two dominant grass species across different pulse rainfall events. J Appl Ecol 54: 997–1007. 10.1111/1365-2664.12816.

[ref74] Lioubimtseva E (2004) Climate change in arid environments: revisiting the past to understand the future. Progress in Physical Geography 28: 502–530.

[ref71] Long RL , Gorecki MJ, Renton M, Scott JC, Colville L, Goggin DE, Commander LE, Westcott DA, Cherry H, Finch-Savage WE (2015) The ecophysiology of seed persistence: a mechanistic view of the journey to germination or demise. Biological Reviews 90: 31–59. 24618017 10.1111/brv.12095

[ref37] Markey A , Kern S, Gibson N (2012) Floristic communities of the Ravensthorpe range, Western Australia. Conservation Science Western Australia 8: 187–239.

[ref38] Merow C , Latimer AM, Wilson AM, McMahon SM, Rebelo AG, Silander JA Jr (2014) On using integral projection models to generate demographically driven predictions of species' distributions: development and validation using sparse data. Ecography 37: 1167–1183. 10.1111/ecog.00839.

[ref73] Merritt DJ , Turner SR, Clarke S, Dixon KW (2007) Seed dormancy and germination stimulation syndromes for Australian temperate species. Australian Journal of Botany 55: 336–344.

[ref39] Mesgaran MB , Onofri A, Mashhadi HR, Cousens RD (2017) Water availability shifts the optimal temperatures for seed germination: a modelling approach. Ecol Model 351: 87–95. 10.1016/j.ecolmodel.2017.02.020.

[ref66] Michel BE (1983) Evaluation of the water potentials of solutions of polyethylene glycol 8000 both in the absence and presence of other solutes. Plant Physiology 72: 66–70. 16662983 10.1104/pp.72.1.66 PMC1066170

[ref72] Miller BP , Symons DR, Barrett MD (2019) Persistence of rare species depends on rare events: demography, fire response and phenology of two plant species endemic to a semiarid Banded Iron Formation range. Australian Journal of Botany 67: 268–280.

[ref40] Murray BR , Thrall PH, Gill AM, Nicotra AB (2002) How plant life-history and ecological traits relate to species rarity and commonness at varying spatial scales. Austral Ecol 27: 291–310. 10.1046/j.1442-9993.2002.01181.x.

[ref41] Onofri A , Benincasa P, Mesgaran MB, Ritz C (2018) Hydrothermal-time-to-event models for seed germination. Eur J Agron 101: 129–139. 10.1016/j.eja.2018.08.011.

[ref42] Onofri A , Mesgaran MB, Ritz C (2022) A unified framework for the analysis of germination, emergence, and other time-to-event data in weed science. Weed Science 70: 259–271. 10.1017/wsc.2022.8.

[ref43] Pascual LS , Segarra-Medina C, Gómez-Cadenas A, López-Climent MF, Vives-Peris V, Zandalinas SI (2022) Climate change-associated multifactorial stress combination: a present challenge for our ecosystems. J Plant Physiol 276: 153764. 10.1016/j.jplph.2022.153764. 35841741

[ref44] Pironon S , Villellas J, Thuiller W, Eckhart VM, Geber MA, Moeller DA, García MB (2018) The ‘Hutchinsonian niche’as an assemblage of demographic niches: implications for species geographic ranges. Ecography 41: 1103–1113. 10.1111/ecog.03414.

[ref45] R Development Core Team R (2022) Rstudio: Integrated Development Environment for r. Rstudio, inc; 2016, Boston, MA

[ref46] Rajapakshe RP , Turner SR, Cross AT, Tomlinson S (2020) Hydrological and thermal responses of seeds from four co-occurring tree species from southwest Western Australia. Conservation Phys Ther 8: coaa021. 10.1093/conphys/coaa021. PMC7192333 32377342

[ref47] Ritz C , Baty F, Streibig JC, Gerhard D (2015) Dose-response analysis using R. PLoS ONE 10: e0146021. 26717316 10.1371/journal.pone.0146021 PMC4696819

[ref48] Ruiz-Talonia L , Carr D, Smith R, Whalley RDB, Reid N (2018) Effect of temperature and light on germination of 10 species of *eucalyptus* from north-western NSW. Australian Journal of Botany 66: 657–666. 10.1071/BT18115.

[ref49] Speißer B , Wilschut RA, Van Kleunen M (2022) Number of simultaneously acting global change factors affects composition, diversity and productivity of grassland plant communities. Nat Commun 13: 7811. 10.1038/s41467-022-35473-1. 36535931 PMC9763497

[ref50] Tomlinson S , Lewandrowski W, Elliott CP, Miller BP, Turner SR (2020) High-resolution distribution modeling of a threatened short-range endemic plant informed by edaphic factors. Ecol Evol 10: 763–777. 10.1002/ece3.5933. 32015842 PMC6988535

[ref51] Tomlinson S , Tudor EP, Turner SR, Cross S, Riviera F, Stevens J, Valliere J, Lewandrowski W (2021) Leveraging the value of conservation physiology for ecological restoration. Restoration Ecology 10: 763–777.

[ref52] Turner SR , Lewandrowski W, Elliott CP, Merino-Martín L, Miller BP, Stevens JC, Erickson TE, Merritt DJ (2018) Seed ecology informs restoration approaches for threatened species in water-limited environments: a case study on the short-range banded ironstone endemic Ricinocarpos brevis (euphorbiaceae). Australian Journal of Botany 65: 661–677. 10.1071/BT17155.

[ref53] Turner SR , Merritt DJ, Renton MS, Dixon KW (2009) Seed moisture content affects afterripening and smoke responsiveness in three sympatric Australian native species from fire prone environments. Austral Ecol 34: 866–877. 10.1111/j.1442-9993.2009.01993.x.

[ref70] Turner SR , Merritt DJ, Ridley EC, Commander LE, Baskin JM, Baskin CC, Dixon KW (2006) Ecophysiology of seed dormancy in the Australian endemic species Acanthocarpus preissii (Dasypogonaceae). Annals of Botany 98: 1137–1144. 17008351 10.1093/aob/mcl203 PMC2803588

[ref54] Turner SR , Steadman KJ, Vlahos S, Koch K, Dixon KW (2013) Seed treatment optimises benefits of seed bank storage for restoration-ready seeds: the feasibility of pre-storage dormancy alleviation for minesite revegetation. Restoration Ecology 21: 186–192. 10.1111/j.1526-100X.2012.00879.x.

[ref55] del Vecchio S , Fantinato E, Roscini M, Acosta AT, Bacchetta G, Buffa G (2020) The germination niche of coastal dune species as related to their occurrence along a sea–inland gradient. J Veg Sci 31: 1112–1121. 10.1111/jvs.12899.

[ref56] Vincent BJ , Barrett S, Cochrane A, Plummer JA, Renton M (2015) Conservation biology of two endemic *beyeria* species (euphorbiaceae) from southern Western Australia. Australian Journal of Botany 63: 484–496. 10.1071/BT14310.

[ref57] Viscarra, Rossel R , Chen C, Grundy M, Searle R, Clifford D, Odgers N, Holmes K, Griffin T, Liddicoat C, Kidd D (2018a) Soil and landscape grid national soil attribute maps - clay (3 resolution) - release 1. Commonwealth Scientific and Industrial Research Organisation (CSIRO).

[ref58] Viscarra, Rossel R , Chen C, Grundy M, Searle R, Clifford D, Odgers N, Holmes K, Griffin T, Liddicoat C, Kidd D (2018b) Soil and landscape grid national soil attribute maps - sand (3 resolution) - release 1. Commonwealth Scientific and Industrial Research Organisation (CSIRO).

[ref59] Viscarra, Rossel R , Chen C, Grundy M, Searle R, Clifford D, Odgers N, Holmes K, Griffin T, Liddicoat C, Kidd D (2018c) Soil and landscape grid national soil attribute maps - silt (3 resolution) - release 1. Commonwealth Scientific and Industrial Research Organisation (CSIRO).

[ref60] Walck JL , Hidayati SN, Dixon KW, Thompson K, Poschlod P (2011) Climate change and plant regeneration from seed. Glob Chang Biol 17: 2145–2161. 10.1111/j.1365-2486.2010.02368.x.

[ref61] Warren RJ , Wright JP, Bradford MA (2011) The putative niche requirements and landscape dynamics of Microstegium vimineum: an invasive Asian grass. Biol Invasions 13: 471–483. 10.1007/s10530-010-9842-4.

[ref62] Yates CJ , Gibson N, Pettit NE, Dillon R, Palmer R (2011) The ecological relationships and demography of restricted ironstone endemic plant species: implications for conservation. Australian Journal of Botany 59: 692–699. 10.1071/BT11199.

