# Supplementary material for: Same, same, but different: dissimilarities in the hydrothermal germination performance of range-restricted endemics emerge despite microclimatic similarities
Source: Conserv Physiol. 2024 Feb 22;12(1):coae009. doi: 10.1093/conphys/coae009 (PMC10939308; doi:10.1093/conphys/coae009)
Supplement: Web_Material_coae009 [file web_material_coae009.zip › Supplementary material FOR RESUBMISSION.pdf]

Same, same, but different: Dissimilarities in the hydrothermal germination performance of range-restricted endemics emerge despite microclimatic similarities.

Supplementary Materials

Rajapakshe P. V. G. S. W. Rajapakshe [1, 2, 3], Sean Tomlinson [4,2†*], Emily P. Tudor[3,5], Shane R. Turner [1,2], Carole P. Elliott [3,5], Wolfgang Lewandrowski [3,5]

[1] ARC Centre for Mine Site Restoration, School of Molecular and Life Sciences, Curtin University, Bentley, WA 6102, Australia.

[2] School of Molecular and Life Sciences, Curtin University, Bentley, Western Australia 6102, Australia.

[3] Kings Park Science, Department of Biodiversity, Conservation and Attractions, Kings Park, Western Australia 6005, Australia.

[4] School of Biological Sciences, University of Adelaide, North Terrace, Adelaide, South Australia 5000, Australia.

[5] School of Biological Sciences, University of Western Australia, Crawley, Western Australia 6009, Australia.

†Current address: Biodiversity and Conservation Science, Department of Biodiversity, Conservation and Attractions, 17 Dick Perry Avenue, Kensington, Western Australia, 6151, Australia

* Corresponding author: sean.tomlinson@dbca.wa.gov.au

ORCID

Subhashi Rajapakshe https://orcid.org/0000-0002-0614-086X

Sean Tomlinson: https://orcid.org/0000-0003-0864-5391

Emily Tudor: https://orcid.org/0000-0002-2628-3999

Shane Turner: https://orcid.org/0000-0002-9146-2977

Carole Elliott https://orcid.org/0000-0003-1744-4911

Wolfgang Lewandrowski: https://orcid.org/0000-0002-7496-7690

Table S1. Distributional extent of the study species according to Australian Virtual Herbarium (https://avh.chah.org.au/).

Species	Area of occupancy in Western Australia (km^2)	Extent of occurrence in Western Australia (km^2)
Tetratheca aphylla **subsp. F.Muell. *aphylla***	16	107
Tetratheca erubescens **J.P.Bull**	0.035	< 2
Tetratheca harperi **F.Muell.**	12	2,412
Tetratheca paynterae **Alford subsp. *paynterae***	40	9,473

Table S2. Collection details (location and date), seed traits (mass, fill and viability) and initial germination following the application of several dormancy alleviation treatments of the four *Tetratheca* species used in this study.

**Species**	**Collection location**	**Collection date**	**Seed Weight (mg)**	**Seed fill (%)**	**Viability (%) of filled seeds (n=15)**	**Germinability (%) of filled seeds* following dormancy alleviation treatments** (n = 15)**				
						Incubation on H_2O for 8 weeks at 15°C.	Incubation on 1µM KAR_1 for 8 weeks at 15°C.	Stratification at 30°C for 4 weeks followed by incubation at 15°C on H_2O for 4 weeks	Stratification at 30°C for 4 weeks followed by incubation at 15°C on 1µM KAR_1 for 4 weeks	Stratification at 30°C for 4 weeks followed by incubation at 20°C on 1µM KAR_1 for 4 weeks
Tetratheca aphylla* subsp. F.Muell. *aphylla	Helena and Aurora Range	Feb. 2019	3.0	79.8 ± 0.1	93.3	0.0***	20.0***	7.1***	26.7***	6.5
***Tetratheca erubescens* J.P.Bull**	Koolyanobbing Range	Feb. 2019	5.1	88.3 ± 1.2	100.0	57.1	46.2	92.9	92.9	80.0
***Tetratheca harperi* F.Muell.**	Mt Jackson Range	Feb. 2019	3.3	96.4 ± 1.7	93.3	57.1	66.7	100.0	78.6	93.3
Tetratheca paynterae* Alford subsp. *paynterae	Windarling Range	Feb. 2019	1.6	83.9 ± 0.2	93.3	42.9	53.8	76.9	92.9	73.3

*Initial germination success of filled seeds was quantified by incubating 15 treated seeds of each taxa on moist germination paper in Petri dishes followed by regular scoring of germination.

**Treatments imposed to alleviate seed dormancy and promote germination (warm stratification and karrikinolide (KAR_1).

***Non germinated seeds were nicked and exposed to 1% (w/v) Tetrazolium (TZ) solution to determine whether non germinated seeds were alive. Embryos in all assessed seeds strongly stained red in the presence of TZ confirming metabolic activity.

Table S3. Mean temperatures (± SE in parentheses) inside seed incubators for the duration of experiments.

**Thermal regime (°C)**	**Temperature (°C)**
10	9.4 (0.02)
15	13.8 (0.01)
20	20.7 (0.03)
22	21.1 (0.02)
25	25.5 (0.01)

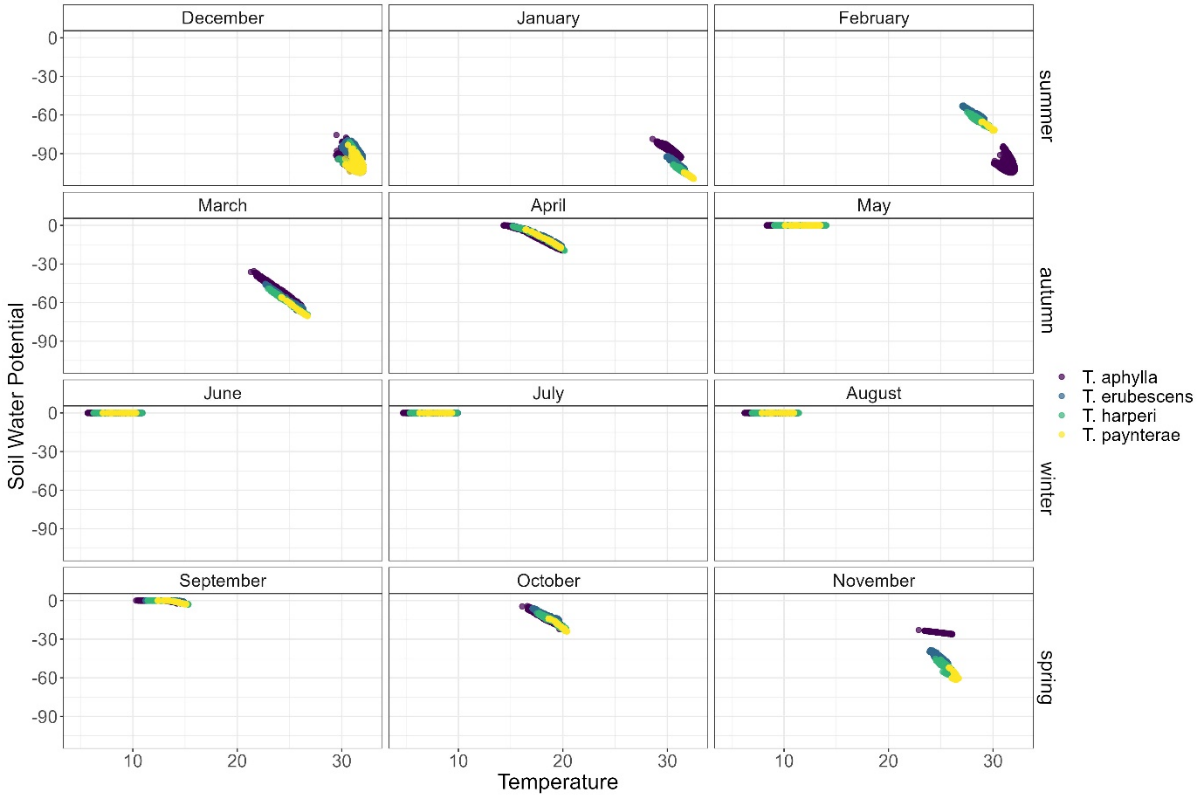

Figure S1: Microclimatic conditions modelled for each known individual of each species at soil depths of 2.5 cm. Locations of most species are characterised by very similar conditions for most of the year, where summers are hot, with very low soil water potentials, but these become very moderate between April and October, across the winter.
